# Structural Characterization and Immunomodulatory Activity of a Novel Polysaccharide From Lycopi Herba

**DOI:** 10.3389/fphar.2021.691995

**Published:** 2021-06-25

**Authors:** Wuxia Zhang, Yihua Hu, Jiaqi He, Dongdong Guo, Jinzhong Zhao, Peng Li

**Affiliations:** Department of Basic Science, Shanxi Agricultural University, Jinzhong, China

**Keywords:** Lycopi Herba, polysaccharide, structure, immunological activity, NF-κB pathway

## Abstract

Lycopi Herba has been broadly used as a traditional medicinal herb in Asia due to its ability to strengthen immunity. However, it is still obscure for its material basis and underlying mechanisms. Polysaccharide, as one of the most important components of most natural herbs, usually contributes to the immunomodulatory ability of herbs. Here, we aimed to detect polysaccharides from Lycopi Herba and examine their potential immunomodulatory activity. A novel polysaccharide (LHPW) was extracted from Lycopi Herba and purified by DEAE-52 cellulose chromatography and G-100 sephadex*.* According to physicochemical methods and monosaccharide composition analysis, LHPW was mainly composed of galactose, glucose, fructose, and arabinose. NMR and methylation analyses indicated that LHPW was a neutral polysaccharide with a backbone containing →3,6)-β-D-Galp-(1→, →4)-β-D-Galp-(1→ and →4)-α-D-Glcp-(1→, with the branches of →1)-β-D-Fruf-(2→ and →6)-α-D-Galp-(1→. Immunological tests indicated that LHPW could activate macrophage RAW264.7 and promote splenocyte proliferation. This study discovered a novel polysaccharide from Lycopi Herba and showed it was a potential immunomodulator.

## Introduction

By virtue of the efficacy and safety, traditional Chinese medicine (TCM) is getting back up to a crucial status in the health area (Zhang et al., 2020). Among them, Lycopi Herba from the dry overground part of the Labiaceae plant *Lyscopus lucidus* Turcz. var. hirtus Regel is a traditional Chinese medicine that is mainly used for promoting blood circulation, removing blood stasis, and strengthening immunity (Lee et al., 2010). Modern pharmacological research displayed that Lycopi Herba possesses a series of activities such as cardiotonic, anti-allergic, anti-inflammatory, anticancer, and antithrombotic activities (Shin et al., 2005; Lee et al., 2008; Kim and Oh, 2018), while it is still unclear which components are mainly responsible for its biological activities. In recent years, some components have been identified for Lycopi Herba, such as luteolin-7-O-β-D-glucuronide methyl ester, phenolic compounds, and oligosaccharides (Lee et al., 2010; Yang et al., 2010; Lu et al., 2018). Among the kinds of components, polysaccharides have received a lot of attention in recent years for their variety of bioactivities, such as immunomodulatory, antitumor, antioxidative, and hypoglycemic activities with lower toxicity and side effects (Kardošová and Machová, 2006; Schepetkin and Quinn, 2006). However, little is known about the polysaccharides of Lycopi Herba for their structures and biological activities.

Here, we aimed to detect polysaccharides from Lycopi Herba and examine their biological activities. As the unique structural features, polysaccharides usually interact with the immune system in the human body and demonstrate immunoregulatory activities. Some polysaccharides such as lentinan have been developed as immunomodulators (Deng et al., 2020). Therefore, we also mainly focused on the immunoregulatory abilities of polysaccharides from Lycopi Herba.

Generally, polysaccharides were extracted from Lycopi Herba and purified by DEAE-52 cellulose chromatography and G-100 sephadex. The monosaccharide composition was analyzed by high-performance liquid chromatography. The polysaccharide backbone was characterized by NMR and methylation analyses. The immunomodulatory ability of polysaccharides was determined by using the phagocytic capacity and lymphocyte proliferation assay. As a result, a neutral polysaccharide LHPW was obtained from Lycopi Herba. The structure of LHPW, including molecular weight, monosaccharide composition, and glycosyl linkages, was systematically characterized. The immunological examination showed that LHPW could activate macrophage RAW264.7 and promote splenocyte proliferation, indicating LHPW might be a potential immunomodulatory agent.

## Materials and Methods

### Herbs and Chemicals

Dried Lycopi Herba was purchased from Bozhou of Anhui Province in China, and identified by the Laboratory of Chinese Medicine, College of Life Sciences, Shanxi Agricultural University. A voucher specimen (No. 20200832) has been deposited at the Laboratory of Functional Polysaccharides, Shanxi Agricultural University. Bovine serum albumin and standard monosaccharides were purchased from Solarbio Science & Technology Co., Ltd. (Beijing, China). Lipopolysaccharide (LPS), 3-(4,5-dimethyl-2-triazolyl)-2,5-diphenyltetrazolium bromide (MTT), trifluoroacetic acid (TFA), and concanavalin A (ConA) were purchased from Sigma-Aldrich. (E)-3-[(4-methylphenylsulfonyl]-2-propenenitrile (BAY 11-7082) was purchased from Beyotime Biotech. All other chemicals were of analytical grade as available.

### Animals and Cells

Kunming mice were purchased from Shanxi Medical University, China. The mice were maintained on a standard pellet diet and water *ad libitum* at 21°C. All experiments were performed under the Regulations of Experimental Animal Administration issued by the State Committee of Science and Technology of the People’s Republic of China. The RAW264.7 cell line was previously preserved in our laboratory. Spleen lymphocytes were obtained by cell strainers and red blood cell lysis buffer.

### Extraction and Purification of Crude Polysaccharide

The dried Lycopi Herba powder was defatted and extracted three times with distilled water at 80°C for 2 h at the ratio of 1:10 (W/V). The supernatants were concentrated and collected with three times alcohol sedimentation. The precipitate was dissolved, dialyzed, and lyophilized to obtain Lycopi Herba crude polysaccharide (LHP). After being purified by DEAE-cellulose column (OH^−^ form) and sephadex G-100 which was eluted with distilled water, polysaccharide LHPW was obtained. Endotoxin contamination was routinely monitored in the laboratory using a chromogenic Limulus Amebocyte Lysate Assay Kit (Xiamen Limulus Reagent Factory, China). LPS existed at a concentration of <0.1 ng/ml in the preparation of LHPW.

### Characterization of Polysaccharide

#### Molecular Weight Determination

As described previously (Zhang et al., 2013), the molecular weight of LHPW was determined by high-performance gel permeation chromatography (HPGPC) on three columns (Waters Ultrahydrogel 250, 1,000, and 2,000; 30 cm × 7.8 mm; 6 µm particles) in series. The columns were calibrated with T-series Dextrans (5.2, 11.6, 23.8, 48.6, 148, 273, and 410 kDa). Sodium acetate (3 mM) was used as eluant, and the flow rate was kept at 0.5 ml/min. 100 μl sample was injected for each run. The calibration curve of log (Mw) vs. elution time (T) is as follows: log (Mw) = −0.1719 T + 11.58.

#### Infrared Spectra Analysis

2 mg purified LHPW was ground with dried KBr and pressed into a pellet for Fourier transform infrared spectrophotometer (BRUKER TENSOR 27, BRUCK, Germany) measurement between 400 and 4,000 cm^−1^ (Zhao et al., 2014).

#### Chemical Composition Analysis

The contents of neutral carbohydrates, proteins, and uronic acids in LHPW were determined by the phenol–sulfuric acid method, Bradford’s method, and *m*-hydroxydiphenyl–sulfuric acid method, respectively. Monosaccharide compositions were analyzed by high-performance anion exchange chromatography (HPAEC) after being hydrolyzed by the 3 M trifluoroacetic acid (TFA) for 2 h (Hu et al., 2020). The hydrolysates were dried by adding 200 μl methanol under nitrogen flow twice, diluted with deionized water, and filtered before injections. A Dionex ICS-5000 (Thermo Scientific Co., Waltham, MA, United States) equipped with a CarboPac™ PA-20 analytical column (3 mm × 150 mm) and an electrochemical detector was employed. Gradient elution with mobile phase NaOH (15 mM) and sodium acetate (100 mM) containing a fixed 15 mM NaOH was employed, and the flow rate was 0.3 ml/min. The column temperature was set at 30°C. Sixteen monosaccharides such as fucose (Fuc), galactosamine (GalN), rhamnose (Rha), arabinose (Ara), glucosamine (GlcN), galactose (Gal), glucose (Glc), N-acetyl-D-glucosamine (GlcNAc), xylose (Xyl), mannose (Man), fructose (Fru), ribose (Rib), galacturonic acid (GalA), guluronic acid (GulA), glucuronic acid (GlcA), and mannuronic acid (ManA), were used as standards. The monosaccharides were identified and quantified by the retention times and corresponding calibration curves of the pure standards. The content of each monosaccharide was expressed as mol% of the total content of monosaccharides.

#### Methylation Analysis

The glycosidic linkages of LHPW were analyzed by methylation analysis based on our previous method with some changes (Zhang et al., 2016). 3 mg dried LHPW was dissolved in 1 ml dried dimethyl sulfoxide (DMSO). After the addition of NaOH, the mixture was sonicated. Methyl iodide (3.6 ml) was added as the methylation reagent, and the mixture was stirred for 60 min at 30°C. The per-methylated product was hydrolyzed, reduced, and acetylated. The partially methylated alditol acetates were analyzed using a gas chromatography–mass spectrometry (GC–MS) system (Shimadzu GCMS-QP 2010) equipped with an RXI-5 SIL MS column (30 m × 0.25 mm × 0.25 μm). The temperature program was started at 120°C, followed by a 3°C/min gradient up to 250°C, isothermal for 5 min. The inlet temperature was 250°C, the detector temperature was 250°C/min, the carrier gas was helium, and the flow rate was 1 ml/min.

#### Nuclear Magnetic Resonance Analysis

50 mg dried LHPW was dissolved in 2 ml of D_2_O and lyophilized. This procedure was repeated three times to completely replace H with D, and the sample was finally dissolved in 0.5 ml D_2_O for NMR analysis. Deuterated acetone was used as the internal reference. The ^1^H NMR, ^13^C NMR, ^1^H-^1^H COSY, HSQC, and HMBC spectra of LHPW were recorded with a Bruker AM 500 spectrometer with a dual probe in the FT mode at room temperature.

### Determination of Immunomodulatory Effects of Polysaccharide

#### Determination of the Phagocytic Capacity and TNF-α Production

RAW 264.7 cells were cultured in 96-well plates (1 × 10^5^ cells/well) and incubated for 24 h. Then cells were treated with various concentrations of LHPW (50, 100, and 200 μg/ml) or LPS (2 mg/ml) for 24 h. The cell supernatants were collected and stored at −80°C. TNF-α proteins were measured using the enzyme-linked immunosorbent assay (ELISA) kits (R&D) according to the instructions. Then 0.5 mg/ml MTT was added to the plates and further incubated for 4 h at 37°C. The optical density was measured at 570 nm. Also, RAW 264.7 cells were pretreated with PBS or BAY 11-7082 (3 µM) for 1 h before incubation with 200 μg/ml or 2 μg/ml LPS for 24 h. Then the TNF-α proteins were measured.

The phagocytic ability of macrophages was measured using neutral red uptake. Cells (1 × 10^5^ cells/well) were pipetted into 96-well plates and treated with various concentrations of LHPW (50, 100, and 200 μg/ml) for 12 h. Then 0.07% neutral red solution was added and incubated for 2 h. The medium was discarded, and cells were washed twice with PBS. Lysis buffer (1% glacial acetic acid: ethanol = 1:1, 100 μl/well) was added, and the optical density of each well was measured at 540 nm. The RMPI1640 medium and LPS (2 μg/ml) were used as the blank and positive control, respectively. The phagocytosis index was calculated by the following equation:

$$\text{Phagocytosis}\;\text{index}=\frac{{\text{Abs}}_{\text{sample}}}{{\text{Abs}}_{\text{blank}\;\text{control}}}.$$ (1)

#### NF-κB Activation and Nuclear Translocation Assay

The RAW 264.7 cells were immunofluorescence-labeled according to the manufacturer’s instruction using a Cellular NF-κB Translocation Kit (Beyotime Biotech) (Xu et al., 2008). Briefly, after washing and fixing, cells were incubated with a blocking buffer for 1 h to block nonspecific binding. Next, cells were incubated with the primary NF-κB p65 antibody for 1 h, followed by incubation with a FITC-labeled Goat Anti-Rabbit IgG (H+L) secondary antibody for 1 h, and then with 4′,6-diamidino-2-phenylindole (DAPI) for 5 min before observation. Finally, p65 protein (green) and nuclei fluoresce (blue) were viewed by laser confocal microscopy.

#### Lymphocyte Proliferation Assay

Lymphocyte proliferation assays were evaluated *in vitro* by the MTT method. The cells (1 × 10^7^/ml) were incubated with different concentrations of LHPW (50, 100, and 200 μg/ml) with or without Con A (5 μg/ml) and LPS (2 μg/ml) for 48 h. MTT was added, and the samples were further incubated for 4 h at 37°C. The optical density was measured at 570 nm.

### Statistical Analysis

Results were expressed as mean ± SD. Statistical analyses were performed with one-way or two-way ANOVA by using the GraphPad Prism 5.0 software. One-way ANOVA analysis was used for the statistical analysis among three or more groups of an experiment with one factor. Two-way ANOVA was used for the statistical analysis of the experiment with two factors.

## Results

### Extraction, Isolation, and Purification of Polysaccharide

**FIGURE 1:**
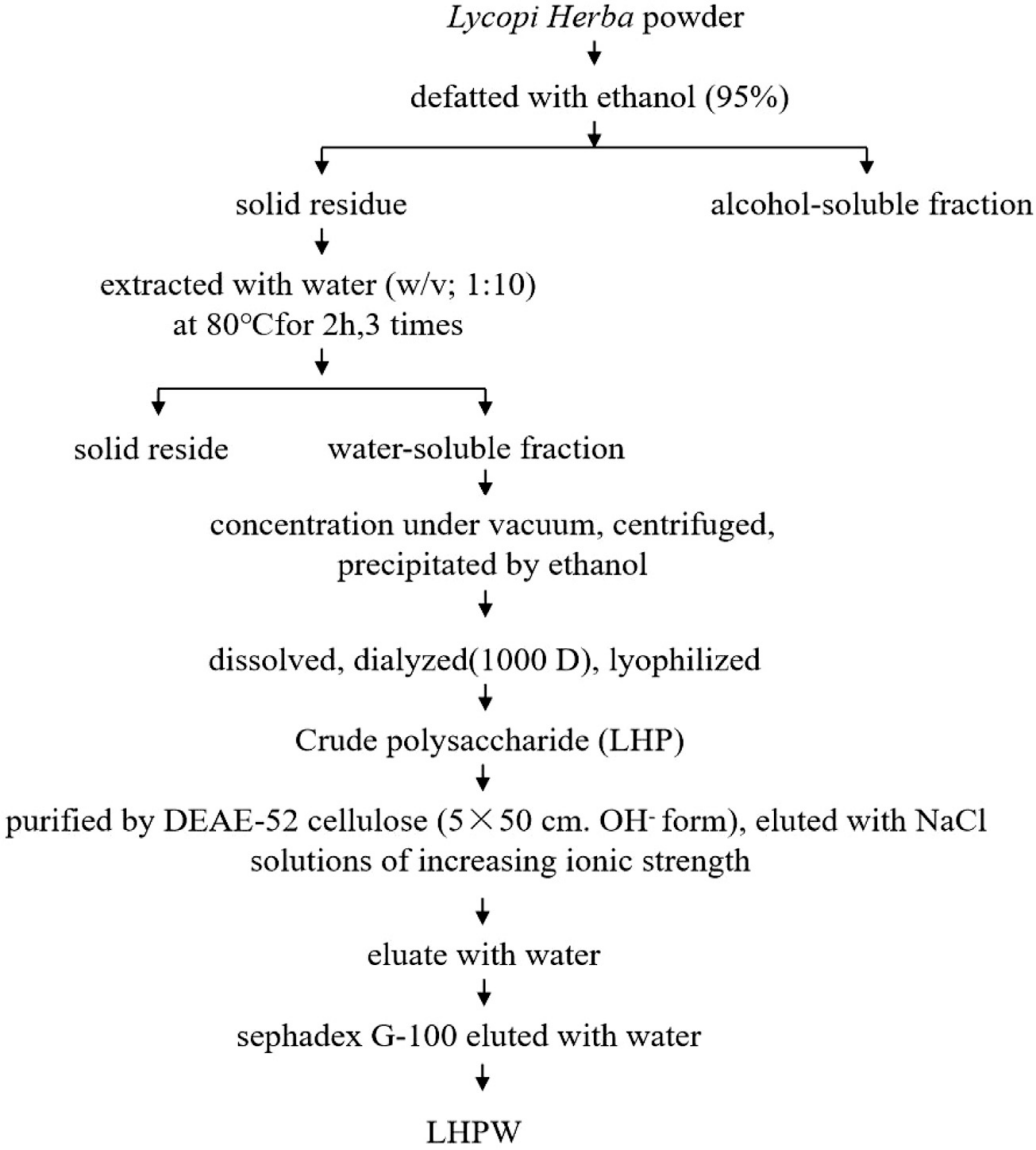
Extraction and purification scheme of polysaccharide LHPW from Lycopi Herba.

The extraction and purification scheme of polysaccharide LHPW is shown in Figure 1. Crude polysaccharide LHP was isolated from Lycopi Herba with a yield of 2.70 ± 0.15%, through hot water extraction, followed by ethanol precipitation and lyophilization. DEAE-52 cellulose column (OH^−^ form) and G-100 sephadex were applied for further purification of the LHP. Finally, a novel heteropolysaccharide designated LHPW was obtained, with the yield of 2.53 ± 0.17% from LHP.

### Molecular Weight Determination

The molecular weight of the polysaccharides is closely related to their physicochemical properties and biological activities (Duan and Kasper, 2011). The molecular weight of LHPW was calculated to be 5,255 Da (T: 45.750 min) in reference to standard dextrans (Supplementary Figure S1A). The symmetrical narrow peak on HPGPC indicated the molecular weight homogeneity and the high purity of LHPW.

### Infrared Spectra Analysis

FTIR spectra of LHPW are shown in Supplementary Figure S1B. A typical absorbance band at 3391.81 cm^−1^ was due to the hydroxyl groups stretching vibration in the constituent sugar residues. The bands in the region of 2932.34 cm^−1^ were the characteristic absorption of C-H stretching vibration in the sugar ring. The absorbance peaks near 1640.32 cm^−1^ suggested that uronic acid content was low in LHPW. The peaks nearby 1,350–1,450 cm^−1^ may be caused by the variable angular vibration of C–H. The peaks at 950–1,200 cm^−1^ suggested the presence of C–O–C and C–O chemical bonds (Kac̆uráková et al., 2000). The peaks nearby 937 and 818 cm^−1^ were the characteristic absorption peaks of furanose.

### Chemical Compositions and Neutral Monosaccharide Composition Analysis

The preliminary chemical composition results showed that the neutral carbohydrate, protein, and uronic acid contents of LHPW were 96.20 ± 2.85%, 4.18 ± 0.34%, and 3.51 ± 0.32%, respectively. These results showed that LHPW was mainly composed of neutral carbohydrates.

**FIGURE 2:**
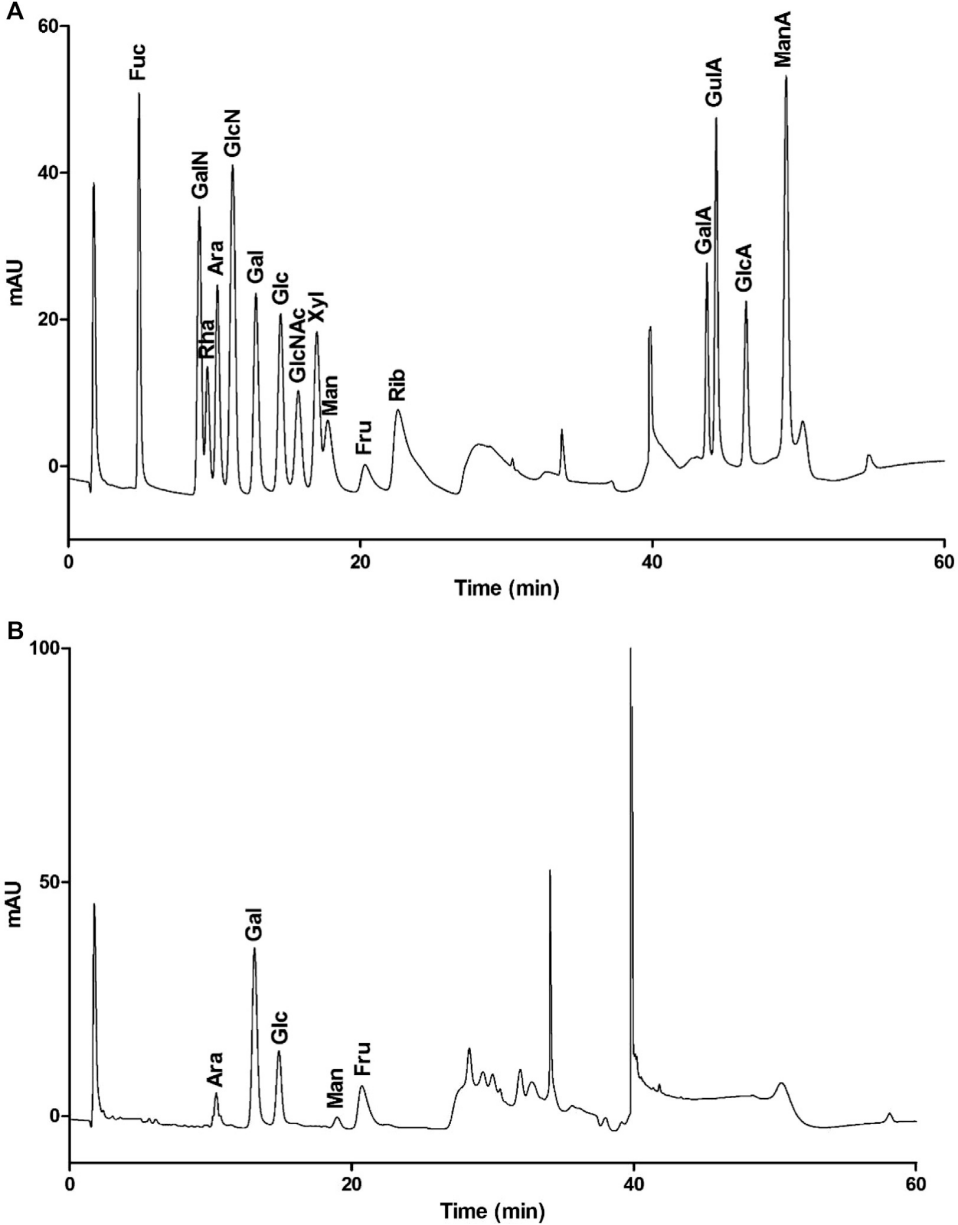
Monosaccharide composition of mixed monosaccharide standards **(A)** and LHPW **(B)**.

To determine the monosaccharide composition, LHPW was hydrolyzed by trifluoroacetic acid, and the hydrolysates were analyzed by high-performance anion exchange chromatography (HPAEC). As shown in Figure 2, the monosaccharide composition analysis indicated that LHPW was mainly composed of galactose, glucose, fructose, arabinose, and mannose at a molar ratio of 48.7:24.2:15.4:10.5:1.2. Galacturonic acid (GalA), glucuronic acid (GlcA), guluronic acid (GulA), and mannuronic acid (ManA) were used as four uronic acid standards. The absence of peaks in the corresponding positions of the samples indicated that there was no uronic acid in the polysaccharide LHPW.

### Methylation Analysis

After two times methylation, the infrared spectra results show that the methylation is complete (Supplementary Figure S2). Methylated LHPW was analyzed by GC-MS in this experiment to elucidate the types of glycosyl linkages (Table 1). The GC-MS chromatogram was included as Supporting Material (Supplementary Figure S3). In the methylation process, 1,2-Fruf were reduced to 1,2-Manp and 1,2-Glcp, so we can obtain the methylated sugar 3,4,6-Me3-Manp and 3,4,6-Me3-Glcp. The total amount of nonreducing terminals was consistent with that of branching residues, suggesting that LHPW was a branched polysaccharide (Chen et al., 2019).

**TABLE 1:** Methylation analysis of LHPW.

No.	Rt	Methylated sugar	Mass fragments (m/z)	Molar ratios	Type of linkage
1	9.375	2,3,5-Me_3_-Araf	43,71,87,101,117,129,161	2.28	Araf-(1→
2	13.033	3,4-Me2-Arap	43,71,87,99,101,113,117,129,161,189	1.53	→2)-Arap-(1→
3	14.538	2,3-Me2-Arap	43,71,87,101,117,129,161,189	1.83	→4)-Arap-(1→
4	16.228	2,3,4,6-Me_4_-Glcp	43,71,87,101,117,129,145,161,205	5.89	Glcp-(1→
5	17.451	2,3,4,6-Me_4_-Galp	43,71,87,101,117,129,145,161,205	4.67	Galp-(1→
6	20.488	3,4,6-Me3-Manp	43,71,87,99,101,129,161,189	4.44	→1)- Fruf--(2→
7	20.828	3,4,6-Me3-Glcp	43,71,87,99,101,129,161,189	7.55	→1)- Fruf--(2→
8	21.07	2,3,6-Me3-Galp	43,87,99,101,113,117,129,131,173,233	6.14	→4)-Galp-(1→
9	21.388	2,3,6-Me3-Glcp	43,87,99,101,113,117,129,131,173,233	9.47	→4)-Glcp-(1→
10	22.213	2,4,6-Me3-Galp	43,87,99,101,117,129,161	1.23	→3)-Galp-(1→
11	22.404	2,3,4-Me3-Glpc	43,87,99,101,117,129,161,189	3.01	→6)-Glcp-(1→
12	22.67	2,3,4-Me3-Manp	43,87,99,101,117,129,161,189	1.38	→6)-Manp-(1→
13	24.407	2,3,4-Me3-Galp	43,87,99,101,117,129,161,189	39.56	→6)-Galp-(1→
14	29.558	2,4-Me2-Galp	43,87,101,117,129,159,189,233	10.02	→3,6)-Galp-(1→

### Nuclear Magnetic Resonance Analysis

**FIGURE 3:**
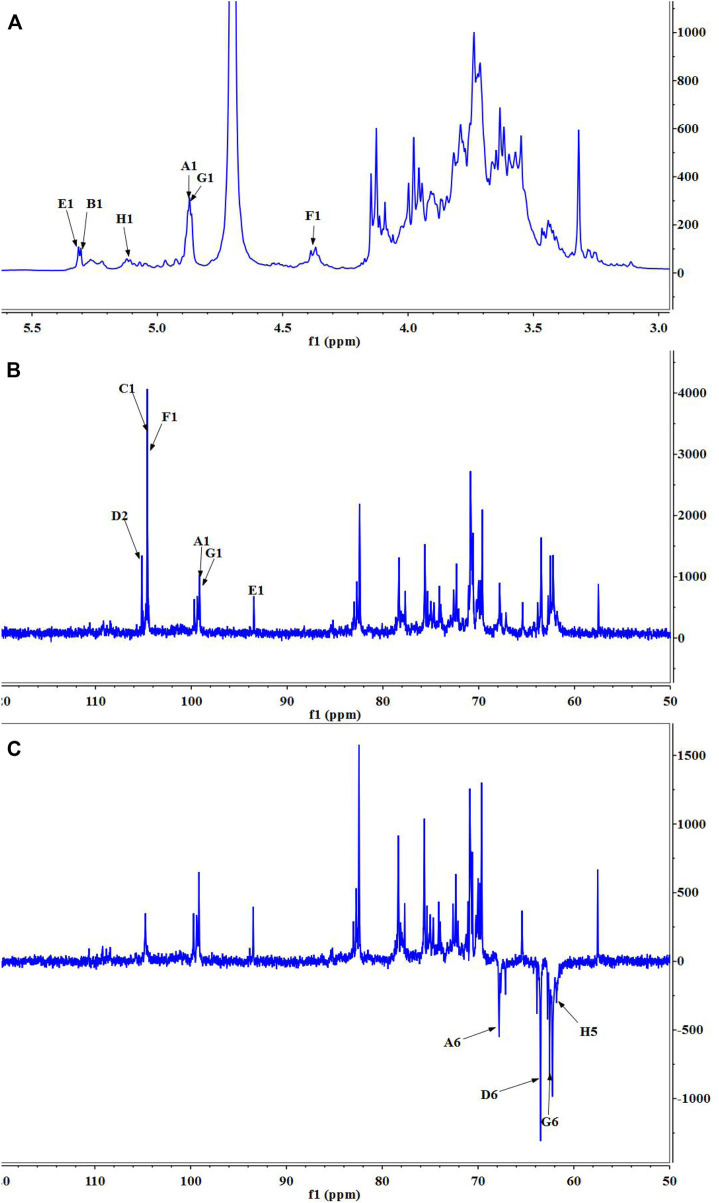
1D NMR spectra of LHPW: **(A)** ^1^H NMR, **(B)** ^13^C HMR, and **(C)** DEPT 135. Same as Table 2, A–H represent the Glycosyl residues →6)-α-D-Galp-(1→, →4)-α-D-Glcp-(1→, →3,6)-β-D-Galp-(1→, →1)-β-D-Fruf-(2→, α-D-Glcp-(1→, →4)-β-D-Galp-(1→, α-D-Galp-(1→, α-L-Araf-(1→. The number 1–6 represent the location of hydrogen or carbon in the Glycosyl residues.

Nuclear magnetic resonance (NMR) spectroscopy was used to reveal the precise structural information of LHPW. The C/H chemical shifts of all monosaccharide residues were assigned as completely as possible and listed in Table 2 based on the above results, 2D NMR spectra, and data available in the literature (Han et al., 2018; Chen et al., 2019). In the ^1^H NMR spectrum of LHPW (Figure 3A), signals at *δ* 4.88, 5.29, 5.32, 4.41, 4.88, and 5.14 ppm corresponded to H-1 of **A**, **B**, **E**, **F**, **G**, and **H**, respectively. The resonances in the region of 90–109 ppm in ^13^C NMR were attributed to the anomeric carbon atoms of LHPW. As shown in Figure 3B, the main signals at *δ* 99.10, 104.69, 93.76, 104.47 99.10, and 105.17 ppm were assigned to the C-1 of **A**, **C**, **E**, **F**, **G**, and C-2 of **D**, respectively. In the DEPT-135 spectra, the inverted signal at *δ* 67.78, 63.47, 62.78, and 62.64 ppm were attributed to C-6 of **A**, **D**, **G**, and C-5 of **H** residues, respectively (Figure 3C). In agreement with the results of monosaccharide composition, there was no absorption peak near 170–180 ppm in ^13^C NMR, which confirms that there was no uronic acid in LHPW (Supplementary Figure S4).

**TABLE 2:** ^1^H NMR and^13^C NMR spectral assignments for LHPW.

Residues	Glycosyl residues	H1a,b	H2	H3	H4	H5	H6a.b	
		C1	C2	C3	C4	C5	C6	
**A**	→6)-α-D-Galp-(1→	4.88	3.76	3.84	4.07	3.98	3.79	3.57
		99.10	69.71	70.73	70.08	70.66	67.78	
**B**	→4)-α-D-Glcp-(1→	5.29	3.53	3.86	3.54	3.75	3.78	ns
		101.14	72.88	74.58	78.35	72.53	61.95	
**C**	→3,6)-β-D-Galp-(1→	4.44	3.54	3.65	4.07	3.84	3.93	3.83
		104.69	71.31	81.5	69.82	74.81	70.76	
**D**	→1)-β-D-Fruf-(2→	3.80,3.60	ns	4.15	3.99	3.75	3.65	3.73
		62.13	105.17	78.3	75.63	82.41	63.47	
**E**	α-D-Glcp-(1→	5.32	3.44	3.65	3.36	3.75	3.71	3.61
		93.76	72.59	73.98	70.59	75.74	61.67	
**F**	→4)-β-D-Galp-(1→	4.41	3.45	3.57	3.96	3.44	3.56	
		104.47	73.21	73.97	79.03	74.36	62.74	
**G**	α-D-Galp-(1→	4.88	3.74	3.84	3.96	4.07	3.79	3.57
		99.1	69.7	70.7	72.52	70.08	62.78	
**H**	α-L-Araf-(1→	5.14	4.1	3.84	4.03	3.73	3.61	
		110.62	82.62	77.97	85.22	62.64		

Note: **A**-**H** represent the Glycosyl residues in the corresponding rows.

The COSY (Supplementary Figure S5A) and HSQC spectrum (Supplementary Figure S5B) were employed to analyze the assignation of other signals in ^1^H NMR and ^13^C NMR. The corresponding heterocephalic hydrogen signal and heterocephalic carbon signal of residue **A** were 99.10 and 4.88 ppm in the HSQC. The cross peaks at δ H/H 4.88/3.76, 3.76/3.84, 3.84/4.07, and 4.07/3.98 ppm were detected in ^1^H–^1^H COSY, which suggested that the signals at *δ* 4.88, 3.76, 3.84, 4.07, and 3.98 ppm corresponded to H-1, H-2, H-3, H-4, and H-5 of the residue →6)-α-D-Galp-(1→ (residue **A**), respectively. According to HSQC, C-1, C-2, C-3, C-4, and C-5 of the residue A were *δ* 99.10, 69.71, 70.73, 70.08, and 70.66 ppm, respectively. The chemical shift of C-6 was 67.78 ppm.

The corresponding heterocephalic hydrogen signal of heterocephalic carbon signal δ104.47 is δ4.41 in the HSQC. The cross peaks at δH/H 4.41/3.45, 3.45/3.57, 3.57/3.96, and 3.96/3.44 ppm were detected in ^1^H–^1^H COSY, which suggested that the signals at δ4.41, 3.45, 3.57, 3.96, and 3.44 ppm corresponded to H-1, H-2, H-3, H-4, and H-5 of the residue →4)-β-D-Galp-(1→ (residue **F**), respectively. Corresponding C-1, C-2, C-3, C-4, C-5, and C-6 were δ104.47, 73.21, 73.97, 79.03, 74.36, and 62.74, respectively. Similarly, the other proton and carbon signals of the rest of the residues were analyzed by the same method.

For fructose residues, the signal at δ105.17 ppm was the typical peak of →1)-β-D-Fruf-(2→, which was assigned to C-2 of fructose. Other carbon signals at 62.13, 78.3, 75.63, 82.41, and 63.47 ppm were correspondingly assigned to C-1, C-3, C-4, C-5, and C-6 of →1)-β-D-Fruf-(2→.

The HMBC spectra (Supplementary Figure S5C) were applied to confirm the glycosyl residues, backbone, and substitution sites of polysaccharides. In the HMBC spectrum, cross peak at δ 104.69/3.96 and δ 104.47/3.96 ppm represented the correlation between C-1 of residue **C** and H-4 of residue **F**, C-1 of residue **F** and H-4 of residue **B**, respectively. Based on the above results, the backbone of LHPW was confirmed by the linkages of →3,6)-β-D-Galp-(1→4)-β-D-Galp-(1→4)-α-D-Glcp-(1→. To further confirm the branches of LHPW, the HMBC spectrum for other residues was analyzed. Cross peaks C-2 and H-1a, b of residue **D** were detected in the HMBC, suggesting the presence of →1)-β-D-Fruf-(2→1)-β-D-Fruf-(2→. In addition, these cross peaks showed the correlation between C-2 of residue **D** and H-6a, b of residue **A**, C-1 and H-6a, b of residue **A**, C-1 of residue **A** and H-6 of residue **C**. The content of other glycoside bonds is relatively small, and no more connection information can be observed in HMBC. Therefore, the glycoside bonds of the main components are analyzed. Based on the above information, the possible structure of polysaccharide LHPW is proposed as Supplementary Figure S6.

### Immunological Activities of Polysaccharide

#### Effects of LHPW on the Phagocytic Capacity and TNF-α Production of RAW 264.7 Cells

**FIGURE 4:**
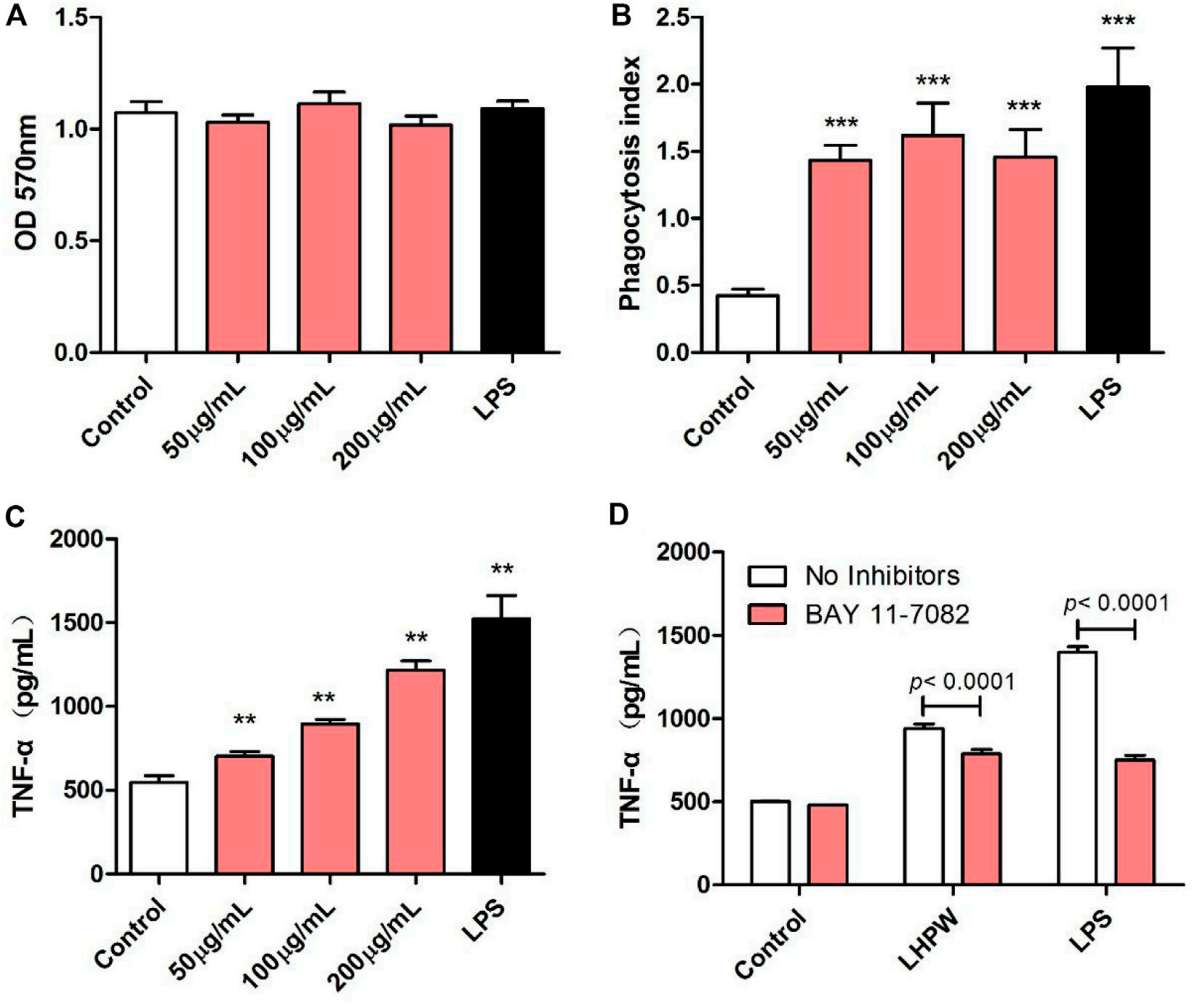
Effects of LHPW on the phagocytosis index, TNF-α production of RAW 264.7 cells. After RAW 264.7 cells were treated with various concentrations of LHPW (50, 100, and 200 μg/ml), the cell cytotoxicity **(A)**, neutral red phagocytosis index **(B),** and TNF-α production **(C)** were tested. Data shown were mean ± SD of three independent experiments. (∗∗∗) *p* < 0.001, (∗∗) *p* < 0.01, and (∗) *p* < 0.05 compared with the control. **(D)** RAW 264.7 cells were pretreated with PBS (No inhibitors) or BAY 11-7082 (3 µM) for 1 h before incubation with 200 μg/ml LHPW or 2 μg/ml LPS for 24 h.

The immunologic action of polysaccharides usually begins with activating major immune cells such as macrophages, natural killer (NK) cells, and lymphocytes (Chen et al., 2012). Macrophages play a very important role in the immune system and perform various complex biological functions such as phagocytosis, surveillance, chemotaxis, and destruction of extraneous materials (Sica et al., 2008). Thus, the effects of LHPW on macrophages were first evaluated. MTT assay showed that LHPW had no cell cytotoxicity to RAW 264.7 cells at different concentrations (Figure 4A). As shown in Figure 4B, LHPW could remarkably enhance the phagocytosis of macrophages. Also, LHPW could significantly promote the TNF-a production of RAW 264.7 cells in a dose-dependent manner (Figure 4C).

Owing to the critical role of NF-κB in the progression of immunity, we detected the effect of LHPW on the activation of NF-κB. To determine whether this NF-κB activation was involved in LHPW-induced cytokine production, we treated RAW 264.7 cells with LHPW and LPS in the presence or absence of BAY 11-7082, a well-described inhibitor of NF-κB. Results showed that treatment with BAY 11-7082 at 3 µM partly suppressed TNF-α secretion induced by LHPW in RAW 264.7 cells (Figure 4D)

**FIGURE 5:**
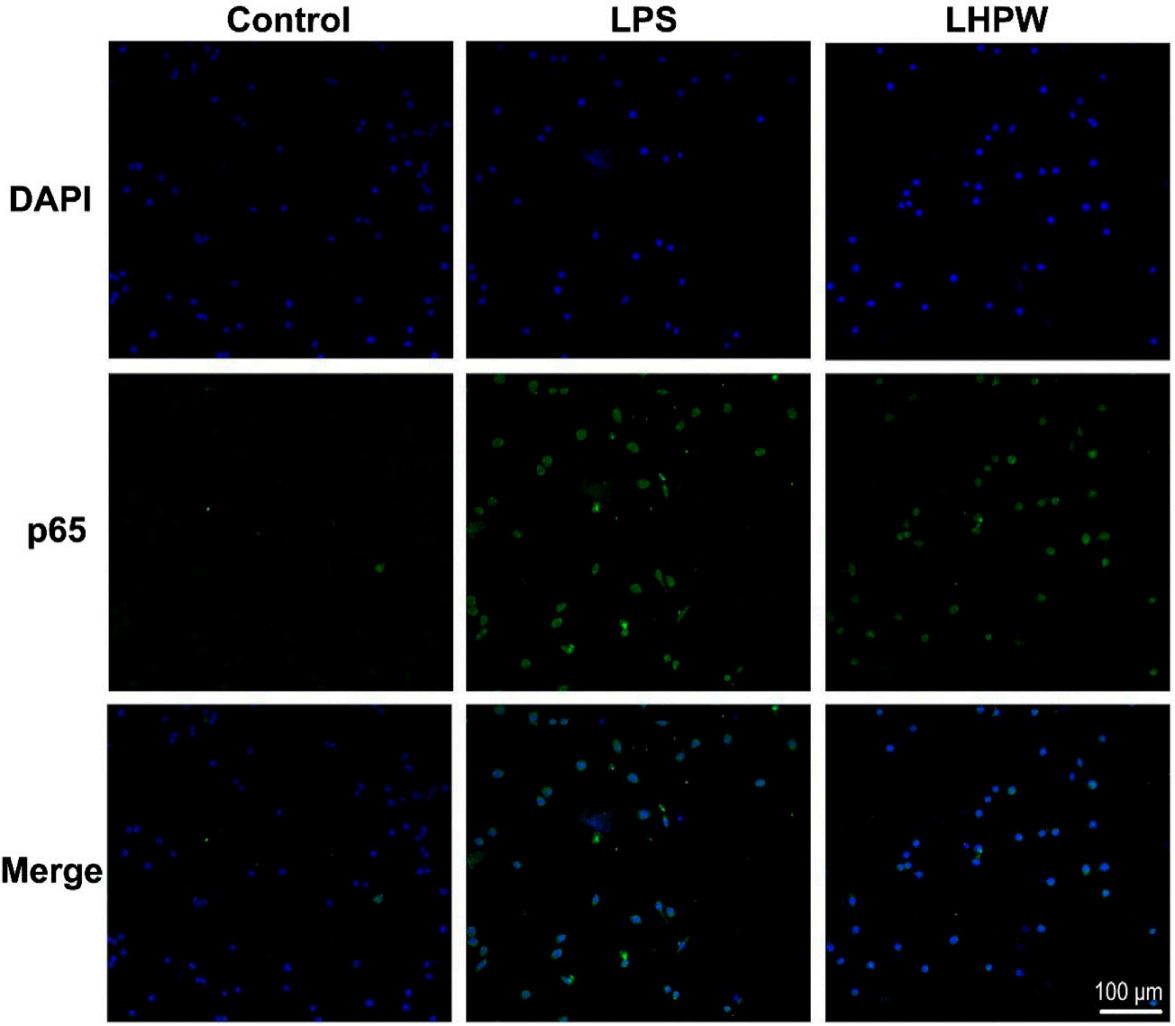
LHPW activates the NF-κB p65 protein in RAW 264.7 cells. After being treated with 200 μg/ml LHPW or 2 μg/ml LPS for 3 h, NF-κB p65 protein translocated into nuclei (DAPI) was obtained by laser confocal microscopy. The scale bar was placed at the bottom right, and all images have the same scales.

By the use of a Cellular NF-κB Translocation Kit, p65 protein translocation into nuclei was visualized in RAW 264.7 cells after exposure to LHPW and LPS. As shown in Figure 5, all stimuli resulted in the localization of the NF-κB transcription factor p65 protein in nuclei, indicating that LHPW could activate the key NF-κB protein p65 in the RAW 264.7 cells.

#### Effect of LHPW on Splenocyte Proliferation

**FIGURE 6:**
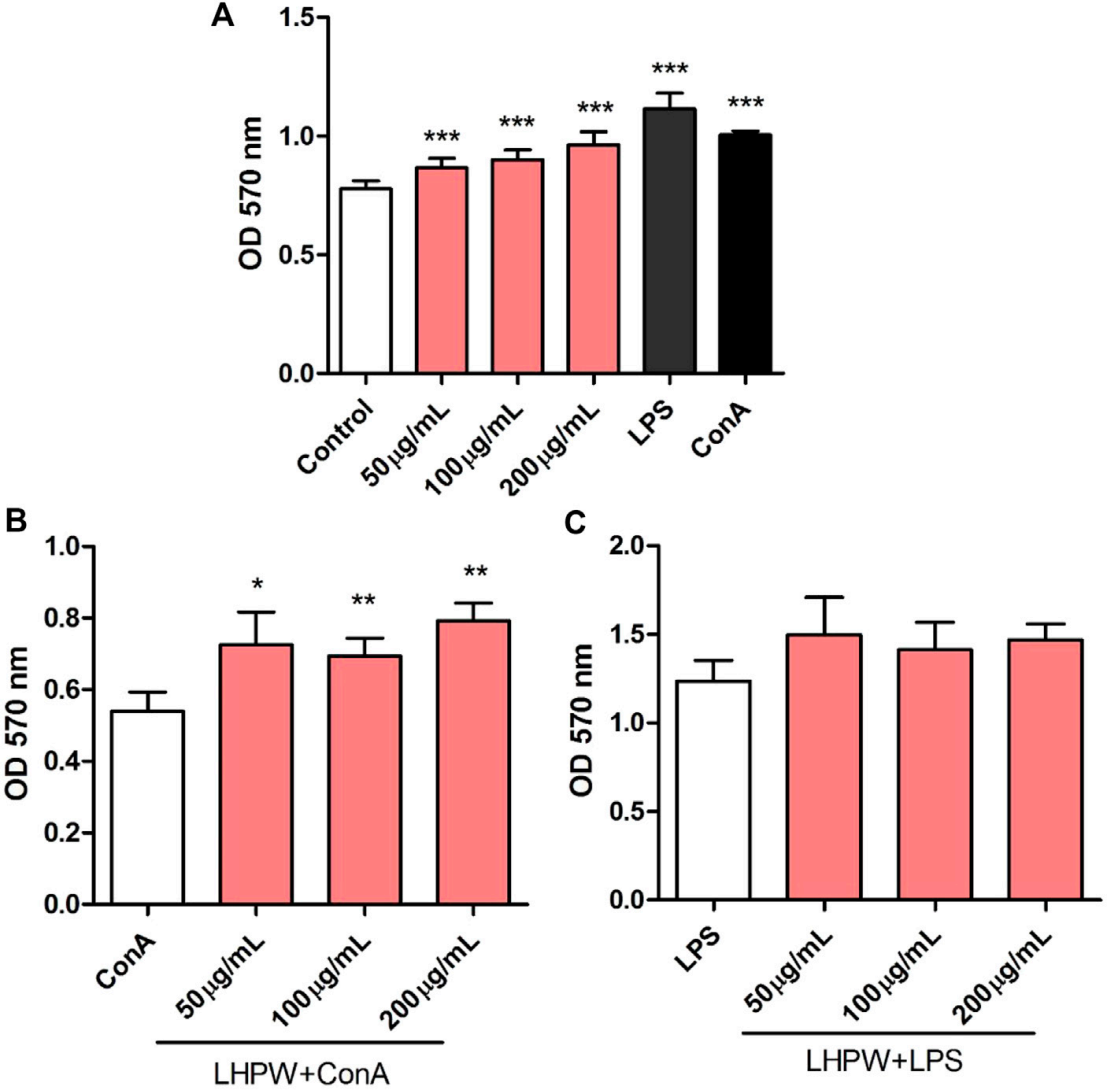
Effects of LHPW on splenocyte proliferation. The mice spleen cells were incubated with LHPW at different concentrations (50, 100, and 200 μg/ml) alone **(A)**, in the presence of mitogens Con A (5 μg/ml) **(B)** or LPS (2 μg/ml) **(C)** for 48 h. MTT was added and incubated. The optical density was then measured at 570 nm. Data were shown as mean ± SD of three independent experiments. (∗∗∗) *p* < 0.001, (∗∗) *p* < 0.01, and (∗) *p* < 0.05 compared with the control group **(A)**, ConA group **(B),** or LPS group **(C)**.

The proliferation of spleen cells is one of the most important steps in the activation pathway of cell-mediated or humoral immunity (Zhao et al., 2006). The effects of the polysaccharide on lymphocyte proliferation were tested to examine the strength of immune-enhancing activity of LHPW. Concanavalin A (ConA) and lipopolysaccharide (LPS) were used to stimulate T-lymphocyte and B-lymphocyte proliferation, respectively (Cerqueira et al., 2004). As shown in Figure 6A, LHPW had the potential in promoting the proliferation of spleen lymphocytes. LHPW also could improve ConA-stimulated T-cell proliferation in a concentration-dependent fashion (Figure 6B). In addition, LHPW could not promote LPS-induced splenocyte proliferation (Figure 6C). Our results indicated that the novel heteropolysaccharide LHPW from Lycopi Herba mainly promoted T-cell–associated lymphocyte proliferation.

## Discussion

In recent years, only some oligosaccharides have been identified from Lycopi Herba. For example, stachyose is a tetrasaccharide composed of “Galactose–Galactose–Glucose–Fructose” and has been reported to have the ability to balance the intestinal micro-ecosystem by selective proliferation of the bifidobacteria. A mixture of α-galactooligosaccharides (GOS) from the roots of Lycopi Herba could elicit a significant increase in humoral immunity, and enhancing in splenocyte proliferation (Yang et al., 2010). However, there are still no bioactive polysaccharides extracted from Lycopi Herba. Here, we aimed to detect potential polysaccharides from Lycopi Herba and explore their structural and biological features.

As a result, a novel water-soluble polysaccharide LHPW with a molecular weight of 5,255 Da was first isolated from Lycopi Herba. The preliminary chemical component analysis indicated that LHPW was mainly composed of neutral carbohydrates (96.20 ± 2.85%). Monosaccharide composition analysis showed that LHPW was mainly composed of galactose, glucose, fructose, and arabinose, and contained no uronic acids. Moreover, ^13^C NMR displayed that there were no peaks near 170–180 ppm, confirming no uronic acids in LHPW. All these results demonstrated that LHPW was a neutral polysaccharide with a backbone containing →3,6)-β-D-Galp-(1→, →4)-β-D-Galp-(1→ and →4)-α-D-Glcp-(1→, with the branches of →1)-β-D-Fruf-(2→ and →6)-α-D-Galp-(1→. The structural characteristics of polysaccharides, such as molecular weight, chemical composition, glycosidic linkage, conformation, and degree of branching, determine their immunomodulating actions (Methacanon et al., 2005). Previously, many studies have reported that neutral polysaccharides have potential as an immunomodulator or supplement in functional food to enhance immunity. For example, a neutral polysaccharide SMP-0b extracted from *Solanum muricatum* could significantly stimulate proliferation and NO production of RAW 264.7 macrophage cells (Yue et al., 2020); a neutral polysaccharide SPW-2, purified from the leaves of *Sambucus adnata* Wall, exerted an immunomodulatory effect by activating macrophages and enhancing the host immune system function (Yuan et al., 2020); a neutral polysaccharide WSRP-1b from *Kushui rose* waste possessed immunomodulatory activity by enhancing phagocytosis of macrophages, increasing the production of ROS, NO, cytokines (IL-6 and TNF-α), and activating the NF-κB signaling pathway (Wu et al., 2020). Inspired by these studies of the immunomodulatory activity of neutral polysaccharides, we also examined the immunomodulatory ability of LHPW through a series of experiments.

The immune response plays an important role in disease development because severe inflammation and suppressed immunization can increase the risk of infections, multiple organ failure, or death (Sharma et al., 2015). It has been reported that many traditional Chinese herbs with the effect of promoting blood circulation and removing blood stasis could also enhance the body’s immune function, which was beneficial to balance immune responses to infection (Fan et al., 2020). To confirm the effect of LHPW on immune response and exposit the molecular mechanism, we used the macrophage RAW 264.7 as induced model cells. Our data indicated that LHPW can enhance the phagocytosis of macrophages, significantly promote the TNF-α production of RAW 264.7 cells, and activate the NF-κB p65 protein translocation into nuclei. Macrophages are immune effector cells that orchestrate a diverse array of functions, including inflammatory response, tissue repair, immune responses, and so on (Jang and Nair, 2013). Thus, our results suggested that LHPW could exert an immune effect by activating macrophages. TNF-α is a potent pro-inflammatory mediator secreted by activated M1 macrophages, which plays a variety of biological effects, such as cell differentiation, proliferation, and multiple pro-inflammatory effects (Wu et al., 2015). Many studies have found that autoimmune-related disorders are correlated with downregulated TNF-α expression (Elenkov and Chrousos, 2002; Russo and Polosa, 2005). Therefore, LHPW which could promote the TNF-α production may have a therapeutic and preventive effect on these diseases. Splenocyte proliferation assay showed it can promote the proliferation of spleen lymphocytes and ConA-stimulated T cells. It should be noted that the immunological study for LHPW here is still preliminary, and more in-depth studies are needed in the following work. Nevertheless, these data implied the novel polysaccharide LHPW from Lycopi Herba had the potential to be a natural immunoregulatory supplement for preparing functional foods and nutraceuticals.

## Data Availability

The original contributions presented in the study are included in the article/Supplementary Material; further inquiries can be directed to the corresponding authors.
