# Supplementary material for: Structural Characterization and Immunomodulatory Activity of a Novel Polysaccharide From Lycopi Herba
Source: Front Pharmacol. 2021 Jun 25;12:691995. doi: 10.3389/fphar.2021.691995 (PMC8267152; doi:10.3389/fphar.2021.691995)
Supplement: Supplementary file 1 [file DataSheet1.PDF]

Structural characterization and immunomodulatory activity of a novel polysaccharide from *Lycopi Herba*

Wuxia Zhang*, Yihua Hu, Jiaqi He, Dongdong Guo, Jinzhong Zhao, Peng Li*

Department of Basic Science, Shanxi Agricultural University, Taigu, 030801, Shanxi, China

*Corresponding author:

Wuxia Zhang: E-mail: wuxia200758@163.com

Peng Li: E-mail: lipengcuc@163.com

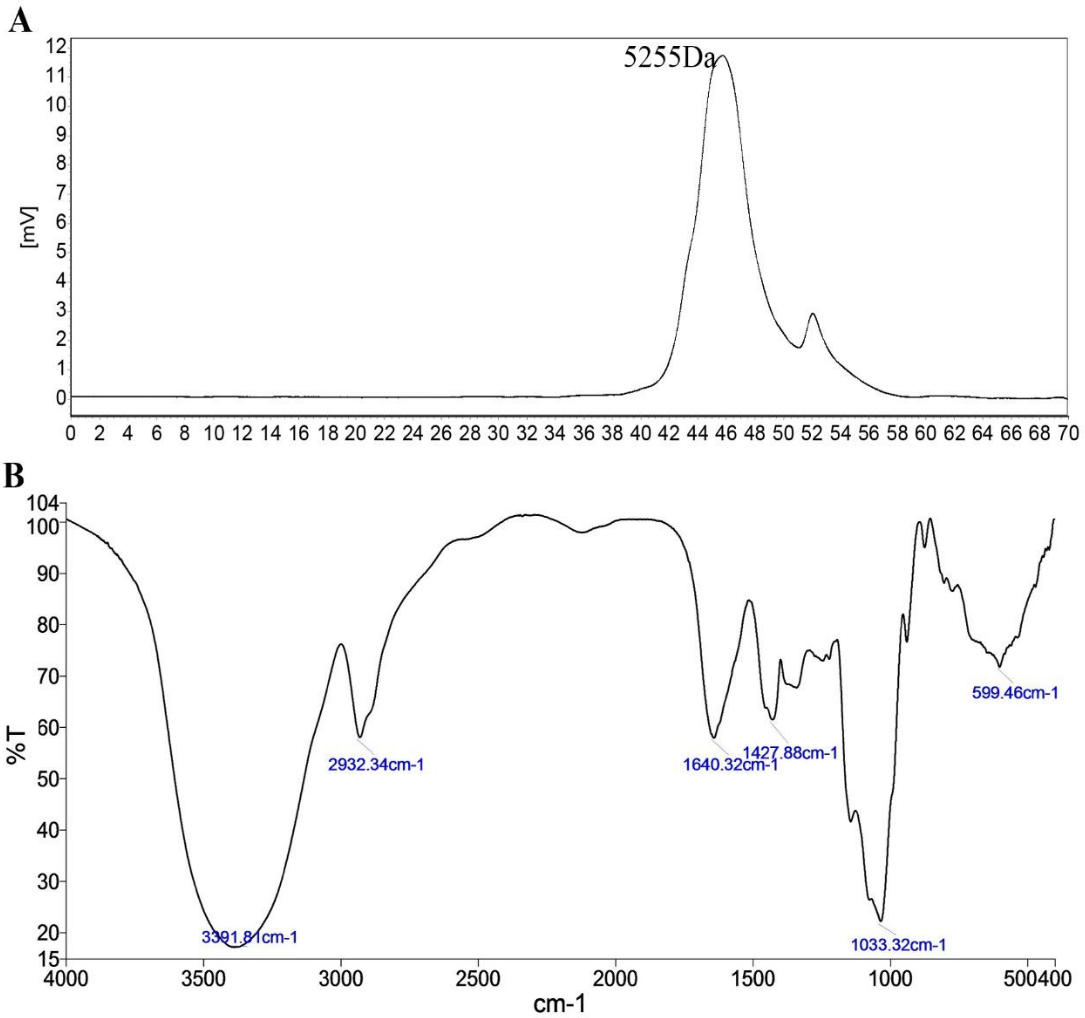

Fig. S1. High Performance Gel Permeation Chromatography spectra of LHPW (A), and Infrared spectra of LHPW (B).

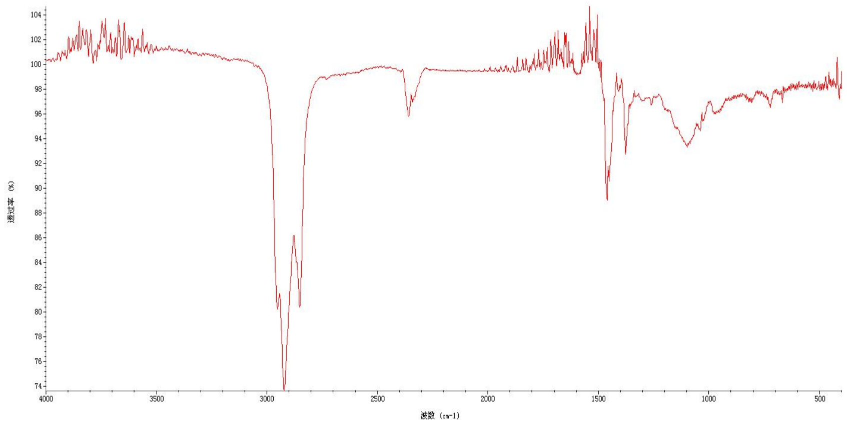

Fig. S2. Infrared spectra of polysaccharide LHPW after two times methylation.

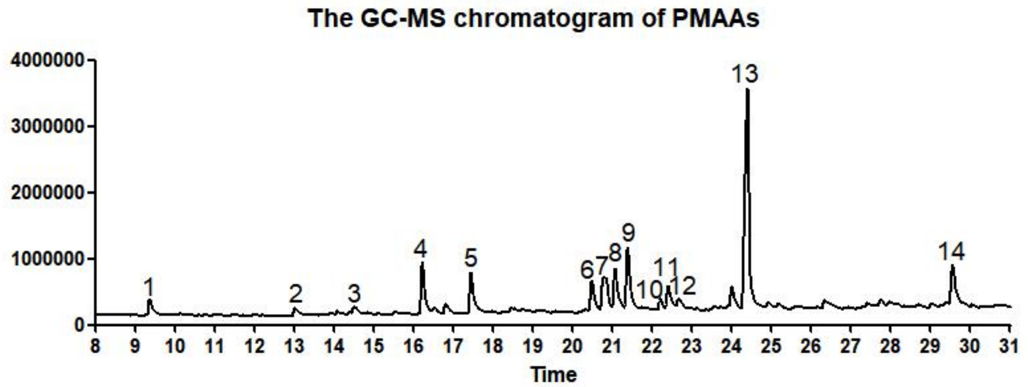

Fig. S3. The GC-MS chromatogram of the partially methylated alditol acetates.

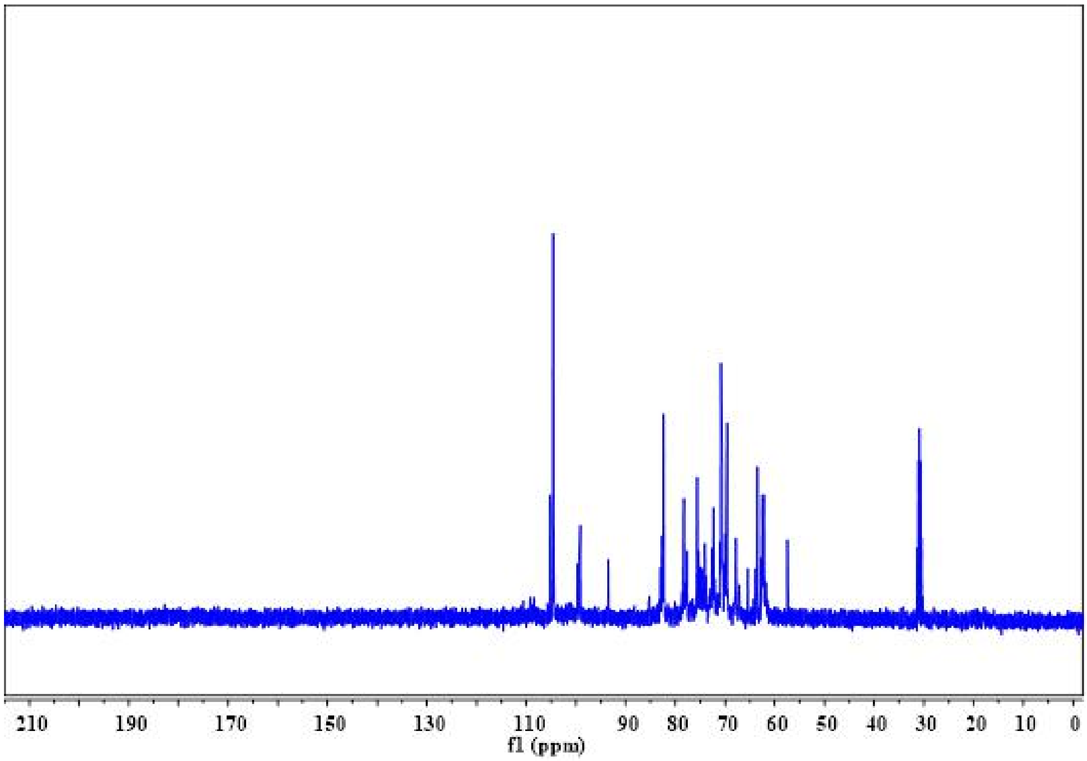

Fig. S4. ^{13}C HMR spectra of LHPW.

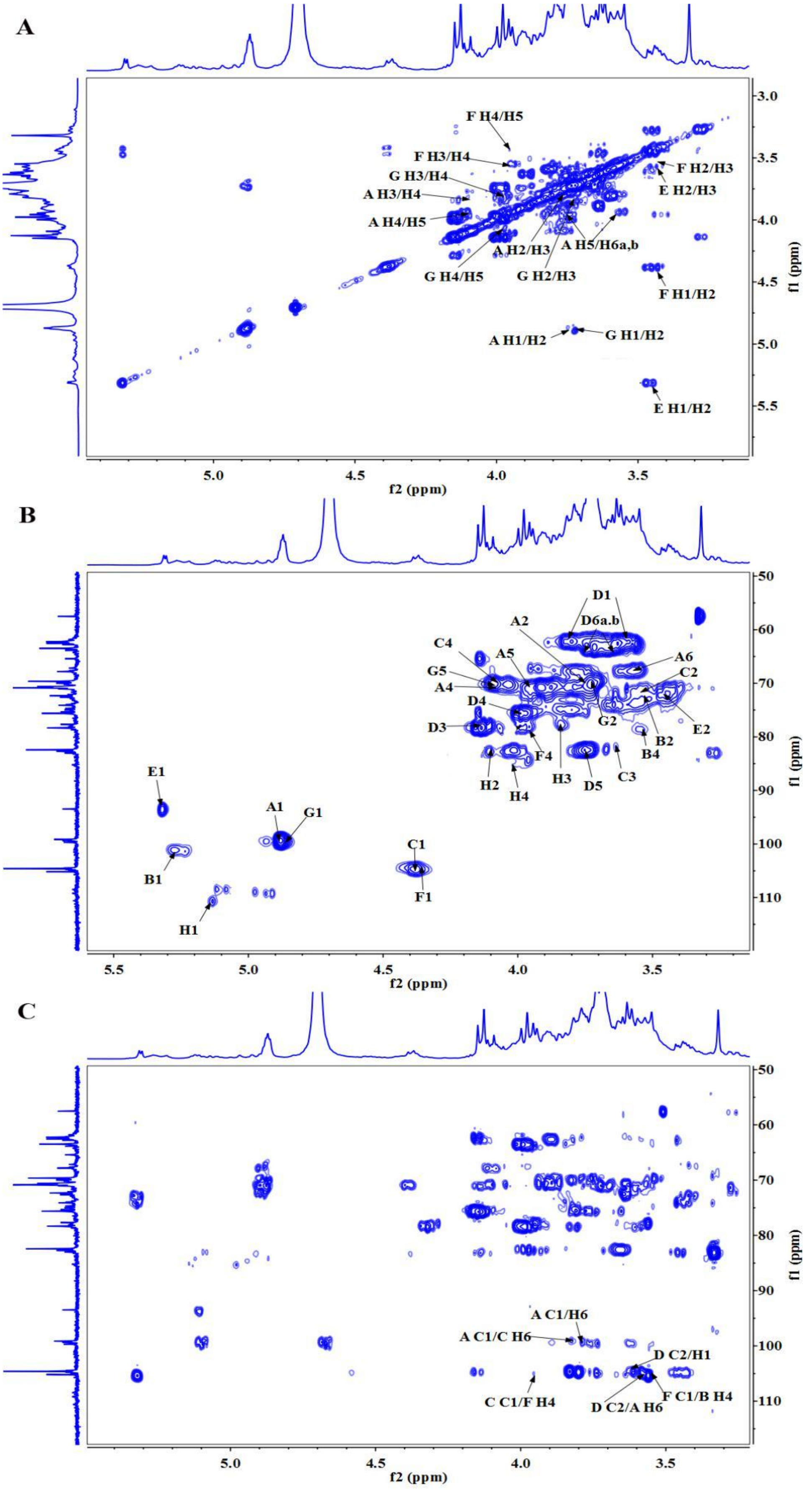

Fig. S5. 2D NMR spectra of LHPW, (A) ^1H-^1H COSY; (B) HSQC; (C) HMBC.

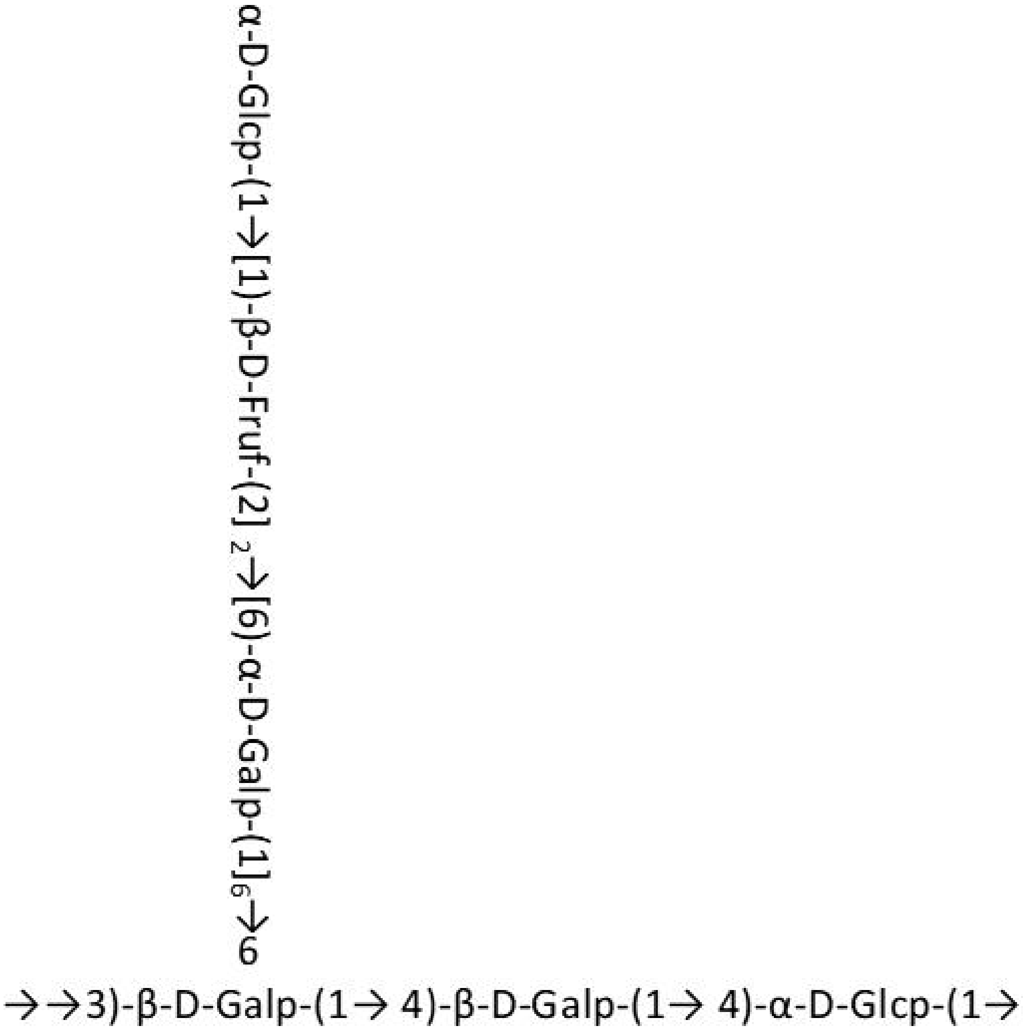

Fig. S6. Putative structure of LHPW.
